# The influence of sociodemographic factors and close relatives at hospital discharge and post hospital care of older people with complex care needs: nurses’ perceptions on health inequity in three Nordic cities

**DOI:** 10.1007/s10433-022-00701-6

**Published:** 2022-04-11

**Authors:** A. E. M. Liljas, N. K. Jensen, J. Pulkki, I. Andersen, I. Keskimäki, B. Burström, J. Agerholm

**Affiliations:** 1 Department of Global Public Health, Karolinska Institutet, Solna, Sweden; 2 Department of Public Health, Copenhagen University, Copenhagen, Denmark; 3 Faculty of Social Sciences, Tampere University, Tampere, Finland

**Keywords:** Ageing, Care system, Healthcare professionals

## Abstract

Hospital discharge of older people in need of both medical and social care following their hospital stay requires extensive coordination. This study aims to examine and compare the views of nurses in three Nordic cities on the influence of sociodemographic factors and having close relatives, for the hospital discharge and post hospital care of older people with complex health and social care needs. Thirty-five semi-structured interviews (Copenhagen *n* = 11, Tampere *n* = 8, Stockholm *n* = 16) with nurses were conducted. The nurses were identified through the researchers’ networks, invitation and snowball sampling, and recruited from hospitals, primary care practices, home care units, home nursing units, and geriatric departments. The interviews were transcribed and analysed using thematic analysis. Interpretations were discussed and agreed within the team. Four main themes and 13 sub-themes were identified. Across the cities, informants reported that the patient’s health status, rather than their gender or ethnicity, steered the discharge date and further care. Care costs, commonly reported in Tampere but also in Copenhagen and Stockholm including costs for medications and home help, were considered barriers for disadvantaged older people. Home situation, local arrangements and differences in collaboration between healthcare professionals at different sites also influenced the hospital discharge. Generally, the patient’s health status steered the hospital discharge and post-hospital care. Close relatives were regarded important and a potential advantage. Some informants tried to compensate for the absence of close relatives, highlighting the importance of care systems that can compensate for this to minimise avoidable inequity.

**Supplementary Information:**

The online version contains supplementary material available at 10.1007/s10433-022-00701-6.

## Introduction

Globally, populations are ageing, increasing the pressure on the healthcare systems (Cristea et al. 2020). Hospital discharge of older adults with complex care needs is a challenging process as it involves multiple activities that requires coordination between several care teams (Mitchell et al. 2010). Studies have shown that poor hospital discharge planning tends to cause delays in communication between healthcare professionals at the hospital, general practitioners and social care providers in the community, which negatively affects the management of the patients (Bull and Roberts 2001; Henwood 2006). Additionally, research has shown that when nurses are exposed to heavy workload, preparing patients and family members for hospital discharge arrangement is not given priority (MacPhee et al. 2017). Yet, the hospital discharge process of older people with complex care needs i.e. those with both medical and social care needs following a hospital stay, requires extensive coordination and is time consuming (Dainty and Elizabeth 2009). Furthermore, close relatives are also known to play an increasingly important role in the hospital discharge process and subsequent social home care (Ulmanen and Szebehely 2015). These concerns raise questions on potential inequity in the hospital discharge process among older adults with complex care needs as healthcare professionals working under pressure sometimes have to make priorities between patients resulting in patients not receiving the right or needed care (Kieft et al. 2014), possibly affecting patients without close relatives worse.

Whilst earlier studies investigating the perceptions of older people, their family members and healthcare professionals on the hospital discharge process have often focused on potential solutions such as the importance of communication and information provided (Bull 1994; Bull and Roberts 2001; Driscoll 2000; Dunnion and Kelly 2007), little attention has been paid to potential inequity. According to Whitehead and Dahlgren (2006), equity in health involves creating opportunities and removing barriers so that everyone can attain their full health potential. To achieve equity in health, the distribution of resources needed for health and access to available opportunities for health need to be fair. There also needs to be fairness in the care offered to people when ill. Additionally, socioeconomic status or other socially determined circumstances should not prevent anyone from achieving their potential health (Whitehead and Dahlgren 2006). Studying health inequity in older populations is important as it undermines healthy ageing (WHO 2017). However, most studies on inequity in the healthcare setting have focused on providing recommendations on how to tackle it with no specific focus on the hospital discharge process (Alvarez-Dardet and Ruiz 2001; Healsip and Nadaf 2019; Woodward and Kawachi 2001). Recent studies on inequity in the hospital discharge have shown that language barriers (Malevanchik et al. 2021) and lack of policy tailored to patients with no fixed address (Jenkinson et al. 2020) contribute to inappropriate discharges and health inequity. However, none of these studies focused specifically on the discharge of older adults.

The hospital discharge planning often starts on admission by hospital-based nurses who also are involved in the preparation of the discharge and the actual transition (most often back home). For older adults with complex care needs who return home, non-hospital-based nurses continue providing post discharge healthcare to them in their homes. Examining the perceptions of nurses on inequity in the hospital discharge process of older people with complex needs is of interest as nurses spend a substantial amount of time with their patients and are therefore likely to bear witness of unequal practices. To our knowledge, no previous study has examined the perceptions of nurses on inequity in the hospital discharge process of older people i.e. differences in the care older people receive due to sociodemographic factors. Considering differences in care due to sociodemographic factors such as gender, ethnicity and socioeconomic status is important to understand how potential inequity might shape the care provided (Bey 2020). Evidently, inequity has to be recognised to be addressed e.g. through policy (European Institute for Gender Equality 2021). Identifying inequity in healthcare is therefore crucial to reduce the gap between social groups and to enable and promote healthy ageing for all older adults. Furthermore, no previous study has obtained data and compared such findings across different comparable welfare systems such as the Nordic systems. The cities compared are all located in welfare states with decentralised systems, yet these decentralised care systems are differently organised suggesting that the findings might be relevant to other decentralised healthcare systems too.

### Aim and research questions

To examine and compare the views of nurses in three Nordic cities on the influence of sociodemographic factors and having close relatives, for the hospital discharge and post hospital care of older patients with complex health and social care needs.

- Research question (RQ) 1: What are nurses’ perceptions of older patients with complex care needs being treated differently due to gender, socioeconomic status and/or ethnic background?
- RQ 2: According to nurses, how may close relatives affect the discharge process of an older family member with complex care needs?
- RQ 3: What similarities and differences are reported on RQ1 and RQ2 in the three cities Copenhagen, Stockholm and Tampere?

### Study context of the hospital discharge

The Nordic countries have built their decentralised health care systems on universal healthcare coverage meaning that all people have the right to the healthcare they need, without financial hardship. Across the countries, delivery of care services is organised at both regional and local levels. Below we give an overview of the hospital discharge processes in the three cities targeted, purposively selected from the Nordic project Social Inequalities of Ageing (sia-project.se): Copenhagen (Denmark), Stockholm (Sweden) and Tampere (Finland). In summary, hospital care is organised at regional level in all three cities. However, in Copenhagen and Tampere, home healthcare and social care are arranged by the municipality whereas in Stockholm home healthcare is provided by the region and social care is provided by the municipality. In Tampere, non-electronic communication such as telephoning between hospital staff and the municipality is necessary as they have separate patient records systems whereas in Copenhagen and Stockholm the hospitals pass on information to the unit delivering the home care using a web-system. Still, all three care systems have the advantage of electronic medical records. Additionally, both healthcare and social care are fully tax-funded in Copenhagen (individuals pay smaller amounts for e.g. rehabilitation and medication) and heavily subsidised in Stockholm (individuals pay service provider costs for healthcare and medications (until paid a total of €112 and then no charges the following 12 months) and smaller amounts for social care too). In Tampere, healthcare is mainly tax-funded (smaller out-of-pocket charges until paid a total of €683 and then no charges in the next 12 months) but individuals pay for social care including post discharge help, costs that can be repaid to people with little money through benefit allowances. More information on the Nordic care systems can be found in the paper “Revisiting the Nordic long-term care for older people—still equal?” (Rostgaard et al. (2022) [under review]) also available in this Issue.

#### Copenhagen

Since 2007 collaboration and coordination of treatment, prevention, discharge and rehabilitation between municipalities and regions have been outlined through mandatory regional healthcare agreements (Olejaz et al. 2012). To ensure the earliest possible involvement of the municipality for citizens in need of care after discharge, the hospital notifies the home care in the municipality about the patient’s care plan, which is a short description of the treatment plan for the patient with a particular focus on the cross-sectional care i.e. care taking provided across the hospital and the primary care sector e.g. when a patient is discharged. The care plan contains information on reasons for admission, expected course of admission, preliminary discharge date, assessed involvement of the municipality, functional ability etc. The care plan must be sent to the municipality within 48 h of admission. Significant changes during admission must be communicated to the municipality as fast as possible (Sundhed 2017). If the patient is considered to be in need of social care, the municipality is notified and an assessment for social services is conducted separately. The hospital department assesses the need for home healthcare upon discharge and, if needed, sends a discharge report to the municipality. The report contains information relevant for the municipality to deliver appropriate care for the patient after discharge and covers areas such as the reason for admission, current level of functioning, what difficulties the patient is experiencing, future appointments with the hospital and points requiring special attention from the municipality (Region Hovedstaden. 2021; Sundhed 2017). Scheduling of the first visit after discharge is based on the professional assessment (Region Hovedstaden. 2021). For citizens already receiving social home care and/or home nursing care before hospitalization the system generates automatic notifications for the municipality and the hospital with basic information. The municipality and hospital can contact each other for additional information through correspondences in an electronically secure system (Sundhed 2017). The patient and their relatives can ask the hospital for a ‘discharge conference’, where hospital staff, assessor from the municipality, the patient and possibly relatives can attend to ensure that the municipality will initiate the necessary assistance after discharge. The hospital is obligated to notify the municipality of the need for assistance even without a discharge conference (Seniorhåndbogen 2021). Since 2015, it has been mandated that patients in need of rehabilitation must receive plan for rehabilitation at the time of discharge at the latest (Sundhed 2017).

#### Tampere

In Finland co-operation is essential to run care services because in many municipalities including the City of Tampere, hospital care is organized by the regional hospital district while community care including primary healthcare, home care and long-term care are organized by the municipality. In Tampere, the hospital district and the city have separate patient records resulting in staff in hospital and home care having to contact each other in person, often by telephoning. Contact ought to be made as soon as the hospital discharge planning starts. It is common that older patients at hospital have been home care clients before the hospitalization and in such cases, nurses from the hospital and home care discuss the patient’s status and care and jointly settle the practicalities regarding the discharge. For clients without preceding home care, physicians may recommend home care and in these cases a discharge counsellor at hospital contacts home care staff and schedule a meeting to arrange for care after discharge. The Health Care Act implies that healthcare professionals are obliged to evaluate whether the patient has a home care need, for example following hospital discharge (Finlex 2016). In addition, co-operation in planning and organizing older patients’ health and social care is outlined in the Act on Older Persons’ Care (Finlex 2012). In cases where the older person has no need for home care after discharge, the older person continues having access to healthcare via the municipality health centre. The City of Tampere also provides a discharge team to help older patients during the first two weeks at home, but for the patient this is a chargeable service and thus not often used (Tampere 2021).

#### Stockholm

In January 2018 a new national law on coordination at hospital discharge (LUS 2017:612) was introduced (Riksdagen 2017). The law was implemented in Region Stockholm in the last quarter of that year. Since then, hospital-based healthcare professionals need to provide an estimated hospital discharge date within 24 h after the patient has been admitted to the hospital. The information is shared with the older person’s primary care clinic responsible for care post hospital discharge, to enable them to allocate a home healthcare nurse to the older person, if needed. Throughout the stay, healthcare professionals at the hospital, typically nurses with care coordination responsibilities, and the home healthcare nurse exchange information about the patient’s health status using a digital computer-based system. Information can also be shared with social care managers who are employed by the municipality. If the older person requires post hospital medical care that is either considerably greater than prior to the hospital stay, or the person has not received home healthcare in the past, the home healthcare nurse arranges for an individual care planning meeting to discuss their needs of health and social care. Whilst such meeting used to take place at the hospital, it is now often arranged for in the older person’s home shortly after the hospital discharge. The home healthcare nurse liaises with the social care manager who attends the meeting if social care seems to be needed. Depending on the older person’s needs, the nurse also liaises with other care professionals such as physiotherapists and psychologists. Close relatives are invited too. If the older person will not need any home healthcare but will need social care following the hospital stay, healthcare professionals at the hospital may liaise directly with the social care manager and arrange for such meeting at the hospital prior to discharge. If there will be no or minor changes to the older person’s current home care arrangements, no meeting needs to be arranged (Riksdagen 2017).

## Methods

### Recruitment and study population

Recruitment and data collection took place in autumn 2018 (October–December) in Copenhagen and Tampere, and in early 2019 (January–April) in Stockholm. Interviews in Stockholm were conducted in 2019 due to the implementation of the new law on care coordination at hospital discharge which commenced in autumn 2018. The delay in data collection was to ensure informants had some experience of and were able to report from the perspective of the new hospital discharge routine.

Study informants were identified through the research team’s wider network, snowball sampling and invitation to departments with predominantly older patients such as geriatrics. The informants were nurses involved in the different phases of the discharge process ranging from hospital care to home healthcare. The job roles and workplaces of the 35 nurses who participated in the study are presented in Table 1. Of the 16 informants from Stockholm, 7 informants were recruited from 6 different general practices (of which 4 are located in disadvantaged areas), and 9 informants from geriatric departments at 7 different hospitals across Stockholm. Of the 11 informants in Copenhagen, four were from home nursing care facilities in the municipality, two were from the central health administration in the municipality and five were from hospital departments. The majority were from disadvantaged areas though the nurses employed in the central administration cover all areas of the city and nurses from one of the selected home nursing care facilities also covered a more affluent area. In Tampere, there is only one hospital with geriatric departments from which we interviewed three nurses. Interviewed home care nurses worked in different areas in the city, with no clear distinction between advantaged and disadvantaged areas.

**Table 1:** Job roles and workplaces of the informants

City	Informant	Job role	Work place
Copenhagen			
C1		Home health care nurse	Home nursing care
C2		Home health care nurse	Home nursing care
C3		Discharge coordinator (nurse)	Municipality of Copenhagen
C4		Discharge coordinator (nurse)	Municipality of Copenhagen
C5		Nurse	Medical department at hospital
C6		Nurse	Medical department at hospital
C7		Follow home nurse	Hospital (several departments)
C8		Coordination consultant (nurse)	Hospital (several departments)
C9		Coordination consultant (nurse)	Hospital (several departments)
C10		Initial assessor (nurse)	Home nursing care
C11		Initial assessor (nurse)	Home nursing care
Stockholm			
S1		Home health care nurse	General practice
S2		Home health care nurse	General practice
S3		Home health care nurse	General practice
S4		Home health care nurse	General practice
S5		Home health care nurse	General practice
S6		Home health care nurse	General practice
S7		Home health care nurse	General practice
S8		Nurse with care coordination responsibilities	Geriatric department at hospital
S9		Nurse with care coordination responsibilities	Geriatric department at hospital
S10		Nurse with care coordination responsibilities	Geriatric department at hospital
S11		Nurse with care coordination responsibilities	Geriatric department at hospital
S12		Nurse with care coordination responsibilities	Geriatric department at hospital
S13		Nurse with care coordination responsibilities	Geriatric department at hospital
S14		Care coordinator (qualified nurse)	Geriatric department at hospital
S15		Care coordinator (assistant nurse)	Geriatric department at hospital
S16		Care coordinator (assistant nurse)	Geriatric department at hospital
Tampere			
T1		Nurse with discharging responsibilities	Geriatric department at hospital
T2		Nurse with discharging responsibilities	Geriatric department at hospital
T3		Nurse with discharging responsibilities	Geriatric department at hospital
T4		Nurse with discharging and care continuity responsibilities	Home care
T5		Nurse with discharging responsibilities	Discharging team
T6		Nurse with discharging and care continuity responsibilities	Home care
T7		Nurse with discharging and care continuity responsibilities	Home care
T8		Nurse with discharging and care continuity responsibilities	Home care

### Interview guide and data collection

The interview guide (Supplementary file 1) was based on previous research. Questions included the informants’ perceptions on priorities when deciding to discharge an older patient, what influences the decision to discharge an older patient, whether some patients are prioritised in terms of staying at hospital or being discharged, and what, if any, are the differences in the discharge process between different groups of older people. Informants were also asked to describe what their job involves and their work part of the discharge process of older patients with complex care needs. The individual, semi-structured interviews were conducted face-to-face in the local language by one researcher in each city (AL, NKJ, JP). All interviews were audio-recorded and transcribed.

### Data analysis

Interview data were analysed using a thematic analysis approach (Braun and Clarke 2006). This approach starts with familiarising with the data: The transcripts were read by at least one researcher in each city (AL, JA, NKJ, JP). Then, themes and sub-themes identified were discussed within the team and descriptions of each theme and sub-theme were outlined. Two interviews in Swedish were coded individually by two researchers (AL and JA) and the minor differences identified were discussed and agreed. The two coded Swedish transcripts were also read by NKJ and JP to establish agreement of the meaning of the codes, reduce the risks of differences in the coding and ensure the coding of the themes would be applied similarly across all transcripts. Themes and sub-themes were then further refined. Two Danish transcripts were then coded independently by NKJ and JA and very few differences were identified. All transcripts were then coded according to the themes and sub-themes agreed. The findings were then discussed in relation to previous literature. The informants have been given the opportunity to read and comment on the manuscript.

## Results

Four themes (underlined) and 12 sub-themes (bold) were identified.

### The potential influence of sociodemographic factors on hospital discharge

When discussing sociodemographic differences between older adults with complex care needs that may influence the hospital discharge process, none of the participants reported that individuals were treated differently due to their **gender**. Similarly, no informant reported that older adults were treated differently by staff due to their **ethnicity**. Among informants in Tampere, older patients from ethnic minority groups were associated with good family support:

“They take better care of their relative. They pick up and transport. That makes it safer to discharge the older person.” (T3)

In Copenhagen and Stockholm, some nurses reported **language barriers** when working with older patients from ethnic minorities. Most of those who reported on language barriers said such barriers were overcome through the involvement of another member of staff speaking that language, or a close relative. Most informants tried to arrange for a professional interpreter when needed, and apart from additional costs to the organisation, the use of an interpreter could have negative consequences on the discharge process for the individual:

“Having to arrange for an interpreter may delay the discharge process.” (S15)

“It’s hard to make it work with interpreters as we need to book them from an external agency. So we try to have some relatives present–I know it is not optimal, particularly if it is younger children… it isn’t.” (C7)

Nurses across all cities reported that **socio-economic status** of the older person such as their educational level or income did not influence the hospital discharge process, but possibly the care post hospitalisation. This was particularly a concern the informants had about very socially marginalised people with limited social network, high alcohol consumption and substance misuse. Informants in all three cities, particularly Tampere where the individual pays a larger amount of the care costs (for which those with low income can be reimbursed) compared to Copenhagen and Stockholm, had experienced older adults declining rehabilitation, medical prescriptions and home care post hospitalisation due to costs.

“There are situations when patients relinquish home care because of costs, those who have really low incomes can receive help from the social worker [to apply for reimbursement].” (T7)

Some informants in Stockholm and Copenhagen reported that they put in more effort to patients with lower socio-economic status:

”[Following hospital discharge] one spends more time on them [patients with lower socio-economic status], I think. It’s everything that has to do with the patient, not just the medical treatment, but contact with the social care manager and so on.” (S7)

“[I can] choose and say I will take him home and spend 3 hours in this house. That might make a difference. … But we are measured by the number of visits, not what type of patient we are dealing with. … To give people the same service, we may need to treat them very differently.” (C7)

A couple of informants from Stockholm also thought that lower socioeconomic status may influence patients’ understanding of the care system, who to contact, and their rights:

“They might know little about the laws and rules and so they struggle.” (S4)

### Close relatives and the discharge process

Informants in all cities reported that relatives play an important role in **providing information** on the patient’s living situation and ability to manage everyday life prior hospitalisation. Close relatives were described as a particularly valuable sources for information of patients with cognitive decline.

“There is no doubt that the network is important. Strong relatives can mean you receive different or further treatment in the hospital. … It also makes a difference after discharge, not in terms of treatment but whether there are any relatives can provide extra information so that we become aware of any issues.” (C4)

”Not having close relatives makes it more difficult to tell what care they need immediately upon return home.” (S2)

“If there are relatives who are strongly involved in the treatment, that will have an impact. One case that comes to mind is a patient who stopped taking his medication [following hospital discharge], but because his daughter was very involved in his treatment, she noticed this. So in that sense patients are better looked after if there is a relative who is very engaged.” (T6)

Most informants in Stockholm and some informants in Copenhagen reported that close relatives play an important role at the discharge process providing practical, psychological and physical **support** by, for example, picking up their older relative at the hospital, and having collected their medications at the pharmacy and filled their fridge with food prior to their return home.

“Having someone to help on the return home prior to the social care home services is up-and-running makes it work easier.” (S2)

“Generally we have a good collaboration with the relatives. There are a lot of patients whose relatives are highly involved and you can easily call and ask if they can do the shopping. They may already have done so. They want to be engaged.” (C5)

All informants in Stockholm and Tampere further reported that they always contact close relatives. In Tampere almost all informants considered being in regular contact with close relatives as part of their job. In Stockholm, the nurses reported contact with close relatives to include inviting them to the individual care planning meeting taking place just before or after the discharge. Some informants also reported that close relatives often take an active role by e.g. helping their older relative explaining what social care is needed to independently manage everyday life post hospital discharge. Informants in Copenhagen also reported contacting close relatives although one of the home healthcare nurses said that this could be improved, but elaborate how organisational issues complicate the involvement:

“We could do that more systematically for the first meeting, formally informing them that a home nurse will be visiting on a certain day and time. … But it is hard to plan. Often I only know the day before that I have a new patient on my schedule.” (C2)

Further, the timing of hospital discharge was reported by a couple of informants in Stockholm to possibly be adjusted slightly according to close relatives’ availability:

“Sometimes their partner who they are dependent on ask ‘Could my husband be discharged in the morning instead of afternoon?’ (S9)

Some informants reported feelings of empathy as well as practical challenges associated with patients with **no close relatives**.

“I feel sorry for those how don’t have any relatives who fight for them when they don’t have the capacity to fight for themselves and tell [the social care manager] what they need and want [following hospital discharge]”. (S8)

“There are unfortunately people without relatives and they are solely dependent on us. … It varies a lot, but there are a lot of lonely people who have no one.” (T5)

Similarly, if a patient has no close relatives to attend the individual care planning, some nurses in all three cities reported that they step in to support the patient by for example emphasizing to the social care manager what the patient asks for in terms of social care, and their healthcare needs:

“I can suggest that advanced healthcare is provided in their home.” (S2)

### Routines and actions preventing inequity

Across the three cities, informants declared that the older individual’s **health and functional status** guided the decision on hospital discharge.

“The only thing that influences when to discharge someone is their medical status.” (S9)

“It’s the physician’s decision when to discharge a patient. … They [patients] are considered ready for discharge as soon as they no longer need a physician around for medical treatment.” (C3)

Most informants in Copenhagen reported that if the patient’s cognitive status affected the patient’s ability to actively participate in the decisions taken related to the discharge process, the system largely compensated for this:

“I physically take the patient home, and then I have a look to see whether the care initiated is appropriate.” (C7)

There were also examples of actions taken by the informants that suggested older adults are **treated equally** irrespectively of gender and ethnicity. Informants in Stockholm reported following existing guidelines, informants in Copenhagen referred to comprehensive checklists to assess level of functional abilities on different aspects of mental and physical health and informants in Tampere used standardised tools to assess the older individual’s abilities.

### Factors and situations where inequity may occur

Postponed hospital discharge due to unfavourable **home situation** was reported by some informants in each city:

“Occasionally we have to keep patients a bit longer until they have had a hospital bed installed in their home. We’ve [also] had a few patients whose homes have been a misery in need of proper sanitation.” (S9)

“Nowadays even a bed-bounded individual can be cared for at home, but that requires a hospital bed and changes to the home environment so that they can still be treated should their condition get worse.” (T4)

Informants in Copenhagen also reported that delays in hospital discharge also applied to patients waiting for a place at a care home:

“If there are no available places, the individual will have to stay at the hospital.” (C4)

Contrary, the hospital discharge went smoother if the home situation was known to the professionals and no major changes were required: “

If they already have home healthcare and social home care then we’ll be able to access their home and that can speed up the return to their home.” (S5)

“Housing can have a great influence on how you manage the situation. One individual might be able to go home whereas it wouldn't work for the other. Even if what they have been through is more or less the same.” (C3)

Some informants in both Copenhagen and Stockholm had experienced **differences in the collaboration between various hospital departments** involved in the care of the patient, which vary between patients and cities due to variation in patient needs and facilities/structure of the care, with consequences to the individual:

“Some departments are really busy. You might not be able to get answers to your questions and so you may need to follow-up on an individual by sending a nurse to the individual’s house the next day [after hospital discharge].” (C11)

“Geriatric departments always contact us before sending someone home but we are not always informed about patients who return home from other hospital departments. Several times patients have returned home without home healthcare being arranged.” (S3)

Because Tampere is a relatively small city, informants collaborate with the same colleagues. However, inequity between patients may appear due to different practices by the nurses as, in the absence of guidelines, the nurses had learnt the steps involved in the discharge process through experience.

“It [the discharge process] works pretty well now that I’ve been here for six years. I’ve gradually figured out who to contact regarding certain matters.” (T4)

In all three cities, informants provided examples of **local arrangements** generating differences in health and social care resources between different geographical areas such as the local municipalities, partly explained by having decentralised care systems. For instance, some areas within Tampere offer home care by physicians in addition to home care provided by a nurse whereas in other areas the patient has to go to their healthcare centre to see a doctor.

“The aim is that over time, all our [older] clients will automatically become doctoral home care clients. But we have so few medical resources that we haven’t been able to do that. Other areas are doing better.” (T8)

The consequences of not getting a home visit by a physician shortly after hospital discharge were discussed by informants in Copenhagen:

“Some GPs are easier to work with than others. Some may not be able to go for a house visit until 14 days post discharge, which is way too long time for the individual, and they can’t go to the GP facility. Then we might need to find another GP for the patient. It isn't easy. The GP has the medical responsibility and all you can do is continue stressing that the patient’s health is worsening.” (C10)

Some local initiatives in Copenhagen and Stockholm were mentioned by the informants and reported to have a positive impact on both healthcare professionals and older adults:

“[The municipality] calls the individual on the day of discharge to inform them about the arrangements and when they will visit. This helps the patient.” (C8)

“Where we are based, patients are offered ‘Safe return home’ which is for those with complex care needs, they get this instead of a bed at a short-term care home. [This involves] a nurse, a physiotherapist and an occupational therapist meeting the patient in their home as they return from hospital to ensure they get the intensive rehabilitation needed to recover.” (S11)

## Discussion

### Summary of results

A summary of the results are provided in Table 2. Informants in the three cities studied reported no differences in the hospital discharge of older adults with complex care needs due to the older individual’s gender and ethnicity per se, though language barriers could delay the discharge process. Differences due to the older individual’s socioeconomic status mainly referred to care costs in the Finnish system, however, in Copenhagen and Stockholm co-payment for e.g. rehabilitation, medications and home help were considered barriers to equity, too. Close relatives were regarded important and a potential advantage in all three cities. Additional factors identified to influence the hospital discharge included the individual’s home situation, poor collaboration and local arrangements. The informants also gave examples of how patients are treated equally and emphasized that the medical status of the patient guided the decision to discharge.

**Table 2:** Summary of results

Issues reported creating differences in the hospital discharge process of older adults	
Gender	None reported (see also discussion)
Ethnicity	No but language barriers could delay the discharge
Socioeconomic status	Yes through charges
Close relatives	A potential advantage
Individual's home situation	Might delay the discharge if changes are needed
Poor collaboration between teams	Might delay the discharge
Local arrangements	Could be both positive and negative

### Sociodemographic factors and the influence of close relatives on equity

The findings suggest that older adults with complex care needs generally tend to be discharged from hospital when the medical treatment that requires hospitalisation is completed irrespective of gender, ethnicity, educational level or income. This is in tension with previous studies showing that higher socio-economic status, being male and being White positively affect the healthcare received (Arpey et al. 2017; Cooper et al. 2017; Pines et al. 2009). Research in psychology has demonstrated that everyday experiences and behaviours among White people are shaped by their privileged position in the social hierarchy (Phillips and Lowery 2018). Similar mechanisms of undervaluing women to the privileging of men have been demonstrated too (Schwiter et al. 2021), suggesting that differences in treatment due to ethnicity or gender might not have been noticed. Also, patients with higher education are likely to have the advantage of communicating more actively, eliciting more information when discussing the discharge plan (Willems et al. 2005). Besides, we did neither ask the informants specifically about their behaviour nor observe them, and we might therefore not have captured the whole picture. Nonetheless, informants considered higher socioeconomic status an advantage if patients are subjected to co-payments of the costs of care, rehabilitation, medications and home help. In the cities studied, people with very low income can apply for financial compensation for care costs, which is particularly applicable to Tampere where older adults are charged for social care yet compensated if having little money. However, research has shown that those in lower socioeconomic groups may not be familiar to, and experience stigma of, claiming benefits available (Braumberg 2016). Furthermore, some people may not qualify for redeemed costs, yet have limited money. Not being able to partly/fully afford social care is sometimes compensated by family members, however the social network of socioeconomically disadvantaged people is often limited (Weyers et al. 2008). Regarding the influence of close relatives, providing information about their older relative’s everyday life and home situation was reported to positively influence the care in all cities. Informants reported that such information from close relatives was particularly important if the older adult was previously unknown to the care teams or was cognitively impaired. This supports studies demonstrating a shift towards patient and family involvement (Lin and Fagerlin 2014; Lindhardt et al. 2008; Nyborg et al. 2017) including a recent systematic review on older adults admitted to hospital emphasizing close relatives’ important role in providing information as it often gets lost in the transfer to hospital (Pulst et al. 2019). These previous studies have, however, not considered the potential risks of inequity including the impact of not having close relatives. Existing literature has however reported on challenges of using family members as interpreters such as incomplete translations and inability to fully grasp the language spoken by staff, making staff feeling stressed and the patient hesitant (Hadziabdic et al. 2010; Seidelman and Bachner 2010). Some informants further initiated responsibility beyond their formal work tasks to compensate for the absence of close relatives. This highlights the importance of care systems that can compensate for lack of relatives. The mix of compassion and work commitment are components previously shown to be crucial in the care sector yet rarely reported on collectively (Sepahvand et al. 2020; Singh et al. 2018). Yet, combined they make the system work though healthcare professionals need to be credited for their extra work or they may lose motivation (Sepahvand et al. 2020).

### Highlights from the findings on how changes to care systems can reduce risks of inequity

This study clarifies that to address health inequity, changes are needed to the wider care system in the form of removing direct costs paid by the older individual (in this study particularly applicable to Tampere), allowing for more time with patients without close relatives, and enabling professionals’ work to involve early and systematic contact making with close relatives to exchange information, particularly if it is the first time the patient will receive care in their home post hospitalisation. A study on the distribution of resources between municipalities in the Nordic countries under review for this Issue has shown that variations in costs do not match variations in need in Finland, and that home help hours are disproportionally fewer in municipalities outside Copenhagen than in the capital region (Rostgaard et al. [under review]). Whilst changes to the care system to avoid inequity related to e.g. costs may require changes to national laws, changes to the healthcare professionals’ work refers to adaptation of their work tasks and strong leadership as they rarely have decision-making roles themselves. For instance, healthcare professionals can do little to delays in getting an interpreter, sanitation of the patient’s home or finding a place at a care home. Such reasons for discharge delays further illustrate the complexity of health equity: addressing them will benefit the patient and potentially reduce inequity between patient groups, yet concurrently rises the possibility of inequity as prolonged hospitalisation increases the risk of infection (Struelens 1998). Additionally, collaboration between professionals involved in the care of the older person has a positive impact on equity through dissemination of patient information, smoother transfers and fewer mistakes, and needs to be facilitated and stimulated on both systemic and interpersonal levels (Maghsoudi et al. 2020).

### Strengths and limitations

Major strengths include that the same interview guide was used across the three cities allowing for cross-country comparison and that the coding was agreed between the researchers. The informants recruited were furthermore involved in different stages of the hospital discharge process. Informants have also commented on the paper, strengthening its trustworthiness. Limitations include nurses as informants being asked about their behaviour and how this may lead to health inequity rather than observed and discrepancies between self-reported and observed behaviour are common (Jenner et al. 2006). This is a study limitation as observations rather than interviews would have provided a better insight into potential inequity caused by the nurses’ behaviour. Further, data were collected from nurses rather than patients on their hospital discharge experience and may therefore present different aspects and consequences of inequality than patients would highlight. Collecting the nurses’ perspectives may have under- or overestimated to what extent inequity is experienced by patients. Limited time and resources prevented us from triangulate the views of nurses with the views of e.g. patients, relatives and hospital/home care managers. Thus, it is possible that some unequal practices have been under- or over-reported and there might also be other issues with inequity than presented in this paper. Further, the study was conducted in two larger cities and one smaller city, possibly affecting the comparability of the cities. Therefore, we have provided detailed information on the three care systems and the methods used to facilitate transparency. Further, data from Copenhagen and Stockholm provided more variations on inequity issues than the data from Tampere. For instance, informants in Tampere reported older patients from ethnic minorities to be rare, restricting their possibility to comment on potential inequity related to ethnicity. However, because the same interview guideline was used, the data are likely to reflect the inequity issues in each care system rather than differences in the data collection. Finally, this study was limited to nurses directly involved in the discharge process and other types of professionals such as physiotherapists and social care managers who sometimes get involved at discharge, were not consulted.

## Conclusions

Older patients’ health and functional status was reported to steer the discharge and post-hospital care. Close relatives were regarded important and a potential advantage. Some informants tried to compensate for absence of close relatives, highlighting the importance of care systems that can compensate for this. Further research should assess the social and financial value of such work undertaken by healthcare professionals beyond their formal work tasks.

## Supplementary Information

Below is the link to the electronic supplementary material.

Supplementary file1 (DOCX 22 KB)

## Data Availability

Anonymised interview transcripts in the original languages can be provided upon request.

## References

[CR1] Alvarez-Dardet C, Ruiz MT (2001). What can doctors do to reduce health inequalities?. J Epidemiol Community Health.

[CR2] Arpey NC, Gaglioti AH, Rosenbaum ME (2017). How socioeconomic status affects patient perceptions of health care: a qualitative study. J Prim Care Community Health.

[CR3] Bey G (2020) Health Disparities at the Intersection of Gender and Race: beyond intersectionality theory in epidemiologic research. In: Quality of life biopsychosocial perspectives. Irtelli, IntechOpen

[CR4] Braumberg B (2016). The stigma of claiming benefits: A quantitative study. J Soc Policy.

[CR5] Braun V, Clarke V (2006). Using thematic analysis in psychology. Qual Res Psychol.

[CR6] Bull MJ (1994). Patients' and professionals' perceptions of quality in discharge planning. J Nurs Care Qual.

[CR7] Bull MJ, Roberts J (2001). Components of a proper hospital discharge for elders. J Adv Nurs.

[CR8] Cooper C, Lodwick R, Walters K, Raine R, Manthorpe J, Iliffe S, Petersen I (2017). Inequalities in receipt of mental and physical healthcare in people with dementia in the UK. Age Ageing.

[CR9] Cristea M, Noja GG, Stefea P, Sala AL (2020). The impact of population aging and public health support on EU labor markets. Int J Environ Res Public Health.

[CR10] Dainty P, Elizabeth J (2009). Timely discharge of older patients from hospital: improving the process. Clin Med.

[CR11] Driscoll A (2000). Managing post-discharge care at home: an analysis of patients' and their carers' perceptions of information received during their stay in hospital. J Adv Nurs.

[CR12] Dunnion ME, Kelly B (2007). Discharge of the older person from the emergency department: the perceptions of health professionals. Int J Older People Nurs.

[CR13] European institute for gender equality (2021) Gender perspective. https://eige.europa.eu/thesaurus/terms/1197 Accessed 20 Dec 2021

[CR14] Finlex (2012) Laki ikääntyneen väestön toimintakyvyn tukemisesta sekä iäkkäiden sosiaali- ja terveyspalveluista [Act on Supporting the Functional Capacity of the Ageing Population and on Social and Healthcare Services for Older People] https://www.finlex.fi/fi/laki/ajantasa/2012/20120980 Accessed 26 Aug 2021

[CR15] Finlex (2016) Terveydenhuoltolaki [Health Care Act]. https://finlex.fi/fi/laki/ajantasa/2010/20101326#L6P53a Accessed 26 Aug 2021

[CR16] Hadziabdic E, Albin B, Heikkilä K, Hjelm K (2010). Healthcare staffs perceptions of using interpreters: a qualitative study. Prim Health Care Res Dev.

[CR17] Healsip V, Nadaf C (2019). Diversity and health inequalities: the role of the practice nurse. Practice Nurs.

[CR18] Henwood M (2006). Effective partnership working: a case study of hospital discharge. Health Soc Care Community.

[CR19] Region Hovedstaden. (2021) Udskrivelse med kommunal sygepleje [Discharge with municipal healthcare]. https://www.regionh.dk/Sundhed/Patientguiden/udskrivelse-fra-hospitalet/Kommunal-sygepleje-og-kommunale-akutteams/Sider/Udskrivelse-med-kommunal-sygepleje.aspx Accessed 14 April 2021

[CR20] Jenkinson J, Wheeler A, Wong C, Pires L (2020). Hospital Discharge planning for people experiencing homelessness leaving acute care: a neglected issue. Healthc Policy | Politiques de Santé.

[CR21] Jenner EA, Fletcher BC, Watson P, Jones FA, Miller L, Scott GM (2006). Discrepancy between self-reported and observed hand hygiene behaviour in healthcare professionals. J Hosp Infect.

[CR22] Kieft RAMM, de Brouwer BBJM, Francke AL, Delnoij DMJ (2014). How nurses and their work environment affect patient experiences of the quality of care: a qualitative study. BMC Health Serv Res.

[CR23] Lin GA, Fagerlin A (2014). Shared decision making: state of the science. Circ Cardiovasc Qual Outcomes.

[CR24] Lindhardt T, Hallberg IR, Poulsen I (2008). Nurses' experience of collaboration with relatives of frail elderly patients in acute hospital wards: a qualitative study. Int J Nurs Stud.

[CR25] Maura MacPhee V, Dahinten FH (2017). The impact of heavy perceived nurse workloads on patient and nurse outcomes. Adm Sci.

[CR26] Maghsoudi T, Cascón-Pereira R, Lara ABH (2020). The role of collaborative healthcare in improving social sustainability: a conceptual framework. Sustainability.

[CR27] Malevanchik L, Wheeler M, Gagliardi K, Karliner L, Shah SJ (2021). Disparities After Discharge: the association of limited english proficiency and post-discharge patient reported issues. Joint Commission J Qual Patient Saf.

[CR28] Mitchell F, Gilmour M, McLaren G (2010). Hospital discharge: a descriptive study of the patient journey for frail older people with complex needs. J Integrated Care.

[CR29] Nyborg I, Danbolt LJ, Kirkevold M (2017). Few opportunities to influence decisions regarding the care and treatment of an older hospitalized family member: a qualitative study among family members. BMC Health Serv Res.

[CR30] Olejaz M, Juul Nielsen A, Rudkjobing A, Okkels Birk H, Krasnik A, Hernandez-Ouevedo C (2012) Health systems in transition. Denmark heath system review. https://www.euro.who.int/__data/assets/pdf_file/0004/160519/e96442.pdf Accessed 14 April 2021 22575801

[CR31] Phillips LT, Lowery BS (2018). Herd Invisibility: the Psychology of Racial Privilege. Curr Dir Psycholl Sci.

[CR32] Pines JM, Russell Localio A, Hollander JE (2009). Racial disparities in emergency department length of stay for admitted patients in the United States. Acad Emerg Med.

[CR33] Pulst A, Fassmer AM, Schmiemann G (2019). Experiences and involvement of family members in transfer decisions from nursing home to hospital: a systematic review of qualitative research. BMC Geriatr.

[CR34] Riksdagen (2017) Lag 2017:612 om samverkan vid utskrivning från sluten hälso- och sjukvård [The law on coordination at hospital discharge]. https://www.riksdagen.se/sv/dokument-lagar/dokument/svensk-forfattningssamling/lag-2017612-om-samverkan-vid-utskrivning-fran_sfs-2017-612 Accessed 14 April 2021

[CR35] Rostgaard T, Jacobsen F, Kröger T, Peterson E ([under review]) Revisiting the Nordic long-term care for older people - still equal? . European Journal of Ageing 10.1007/s10433-022-00703-4 PMC9062627 35528216

[CR36] Schwiter K, Nentwich J, Keller M (2021). Male privilege revisited: how men in female-dominated occupations notice and actively reframe privilege. Gend Work Organ.

[CR37] Seidelman RD, Bachner YG (2010). That I won't translate! experiences of a family medical interpreter in a multicultural environment. Mt Sinai J Med.

[CR38] Seniorhåndbogen (2021) Hjælp ved udskrivning fra sygehus [Help at hospital discharge]. https://seniorhaandbogen.dk/sundhed/sygdom/hjaelp/ Accessed 14 April 2021

[CR39] Sepahvand F, Mohammadipour F, Parvizy S, ZagheriTafreshi M, Skerrett V, Atashzadeh-Shoorideh F (2020). Improving nurses' organizational commitment by participating in their performance appraisal process. J Nurs Manag.

[CR40] Singh P, Raffin-Bouchal S, McClement S, Hack TF, Stajduhar K, Hagen NA, Sinnarajah A, Chochinov HM, Sinclair S (2018). Healthcare providers' perspectives on perceived barriers and facilitators of compassion: results from a grounded theory study. J Clin Nurs.

[CR41] Struelens MJ (1998). The epidemiology of antimicrobial resistance in hospital acquired infections: problems and possible solutions. BMJ.

[CR42] Sundhed (2017) Kommunikation mellem sygehuse og kommuner [Communication between hospitals and municipalities]. https://www.sundhed.dk/sundhedsfaglig/information-til-praksis/syddanmark/almen-praksis/it/elektronisk-kommunikation-tvaersektoriel/kommuner/kommunikation-sygehuse-kommuner/ Accessed 14 April 2021

[CR43] Tampere (2021) Kotiutustiimi -kotisivu [Home page for discharge team]. https://www.tampere.fi/sosiaali-ja-terveyspalvelut/terveyspalvelut/sairaalat-ja-poliklinikat/kotiutustiimi.html Accessed 26 August 2021

[CR44] Ulmanen P, Szebehely M (2015). From the state to the family or to the market? Consequences of reduced residential eldercare in Sweden. Int J Soc Welfare.

[CR45] Weyers S, Dragano N, Möbus S, Beck E-M, Stang A, Möhlenkamp S, Jöckel KH, Erbel R, Siegrist J (2008). Low socio-economic position is associated with poor social networks and social support: results from the heinz nixdorf recall study. Int J Equity Health.

[CR46] Whitehead M, Dahlgren G (2006) Concepts and principles for tackling social inequalities in health. https://www.euro.who.int/__data/assets/pdf_file/0010/74737/E89383.pdf Accessed 26 June 2021

[CR47] WHO WHO (2017) Global Strategy and Action Plan on Ageing and Health (2016–2020). http://who.int/ageing/GSAP-Summary-EN.pdf Accessed 14 April 2021

[CR48] Willems S, De Maesschalck S, Deveugele M, Derese A, De Maeseneer J (2005). Socio-economic status of the patient and doctor-patient communication: does it make a difference?. Patient Educ Couns.

[CR49] Woodward A, Kawachi I (2001). Why should physicians be concerned about health inequalities? Because inequalities are unfair and hurt everyone. West J Med.

