# Supplementary material for: The influence of sociodemographic factors and close relatives at hospital discharge and post hospital care of older people with complex care needs: nurses’ perceptions on health inequity in three Nordic cities
Source: Eur J Ageing. 2022 Apr 11;19(2):189–200. doi: 10.1007/s10433-022-00701-6 (PMC9156630; doi:10.1007/s10433-022-00701-6)
Supplement: Supplementary file 1 — Supplementary file1 (DOCX 22 KB) [file 10433_2022_701_MOESM1_ESM.docx]

Interview questions for nurses

Briefly describe what your job involves including your work tasks related to the discharge process of older adults.

Please describe the process older adults with complex health needs and in great need of health and social care face when discharged from hospital.

-who is involved in such process?

-how do these people/units interact?

-what are the priorities when deciding to discharge an older person?

-when prioritising, who may stay/be discharged?

-do people sometimes get discharged too early? Why?

-what are the differences between those prioritised to stay versus sent home or to care homes? (Health status? Resources needed (incl. staff)? Age? Gender? Family influence/support? Patient’s living arrangements own home/care institution?)

-are there any differences in the process depending on a) ethnical background or citizenship of the patient, b) gender, c) socio-economic position? (why? Examples?)

-what is done to involve the patient/take the patient’s views into account in the decision making and provision of care?

-what is done to maximise a safe return home after discharge?

-do you follow a routine for this or do you treat every case differently? (to what extent do you do so?)

-is the discharge process targeted and adapted to certain groups of patients (e.g. older, certain needs)?

-besides the need for medical treatment, what influences the decision to discharge a patient? (financial incentives?)

-who does the follow up of the patient’s health once the patient has returned home? (when?)

-what does the collaboration between hospital, primary care and social care look like?

-how is contact between hospital, primary care and social care established and undertaken?

-what is done to keep hospital, primary care and social care connected?

-what is done to involve the patient in the decision making and provision of care?

-what is done to prevent readmission to hospital?

-who is responsible for preventing readmission? (What could be improved?)

Anything to add?
